# Measurement of doctor wellbeing prior to the Covid pandemic: a methodological systematic review

**DOI:** 10.1093/occmed/kqaf088

**Published:** 2025-10-06

**Authors:** G Simons, D Opalinski, J Jenkins, E Boxley, D S Baldwin

**Affiliations:** Centre for Workforce Wellbeing, University Department of Psychiatry, University of Southampton, Southampton SO14 3DT, UK; Clinical and Experimental Sciences, Faculty of Medicine, University of Southampton, Tremona Road, Southampton, Hampshire, SO166YD, UK; Hampshire Isle of Wight Healthcare NHS Foundation Trust, Neurological Rehabilitation, Western Community Hospital, Willaim Macleod Way, Southampton, Hamphsire, SO16 4XE, UK; St Mary’s Hospital, Imperial College Healthcare NHS Trust, Praed Street, London, W2 1NY, UK; St Georges University Hospitals NHS Foundation Trust, London SW17 0QT, UK; Clinical and Experimental Sciences, Faculty of Medicine, University of Southampton, Tremona Road, Southampton, Hampshire, SO166YD, UK; Clinical and Experimental Sciences, Faculty of Medicine, University of Southampton, Tremona Road, Southampton, Hampshire, SO166YD, UK; University Department of Psychiatry and Mental Health, University of Cape Town, Cape Town, South Africa

## Abstract

**Background:**

There is no consensus definition of wellbeing, yet it is a key outcome for workforces.

**Aims:**

To describe which wellbeing outcomes had been measured in doctors and which wellbeing outcome measurement instruments had been used with doctors.

**Methods:**

A methodological review of existing literature. MEDLINE, EMBASE, Cochrane Central Register of Controlled Trials (CENTRAL), PsycINFO and the International Bibliography of Social Science were searched for all study types, in all languages. Wellbeing outcomes were categorized as being operationalized in the aims, methods or results and by whether the outcome used to represent wellbeing included the word wellbeing, another positive concept, a pathological symptom, a pathology and were work- or doctor-specific. The outcome measurement instruments used were then categorized and the frequency collected.

**Results:**

A total of 218 studies were included in this review. The total number of unique outcomes used to capture wellbeing in the eligible studies was 57, with 369 non-unique outcomes. Two hundred and fifty-eight of the outcomes used contained the word wellbeing, its components and other positive concepts. For the outcome ‘general wellbeing’ alone, 92 different measurement tools were used. The Maslach Burnout Inventory was the most frequently used measurement tool for all outcomes and was used in 34 studies.

**Conclusions:**

Wellbeing has been measured heterogeneously in doctors in terms of the outcomes and the outcome measurement instruments used. In approximately one-third of the times it was measured, the best that could be achieved was an absence of pathological symptoms, as a negative concept operationalized it.

Key learning points **What is already known about this subject:**

- ‘Wellbeing’ has no international consensus definition.
- Pathological measurement instruments are often used to describe wellbeing.
- A systematic review is normally undertaken to identify possible outcomes and outcome measurement instruments for a Delphi consensus to address these issues.

**What this study adds:**

- A novel categorization was designed of published conceptualization of poorly defined concepts.
- Studies measured wellbeing heterogeneously; the outcomes and measurement instruments used.
- Wellbeing was measured as a negative concept in 30% of studies. The best that could be achieved was an absence of pathology; thriving was not sought or captured.

**What impact this may have on practice or policy:**

- Using self-created wellbeing outcome measurement instruments hinders knowledge and practice progress.
- Meta-analysis of wellbeing interventions will not be possible until appropriate outcomes and measurement instruments are used.
- A minimum agreed set of outcomes, a core outcome set, and recommended measurement instruments would allow comparisons across studies and changes practice.

## INTRODUCTION

‘Wellbeing’ has no international consensus definition [1], and inconsistent approaches to defining wellbeing in the literature have led to use of a diverse range of outcomes [2,3]. A systematic review of wellbeing measurement scales identified 60 different subjective wellbeing measurement tools [2], and a more recent systematic review identified 99 measures [3]. In the UK, policy documents on doctor wellbeing, from the British Medical Association (BMA) [4–6], Society of Occupational Medicine [7], General Medical Council (GMC) [8] and Health Education England [9] that describe and make recommendations on doctors’ wellbeing, share the same lack of operationalization of wellbeing. In a systematic review, only 11 of the 78 included papers contained an explicit definition of doctor ‘wellness’ [10].

There are multiple factors to consider in the measurement of wellbeing. These include why it is being measured: as examples, for screening of pathology; describing epidemiology; or assessing the efficacy of an intervention. Who measures also matters—an independent, impartial, third-party doing the measuring could be verified and is ‘objective’. However, subjective measurement is used most often for wellbeing, where answers can be ranked by the individual with numbers to make ordinal data. The locale of the measurement of wellbeing is relevant; it could be measured nationally (as examples, the NHS staff survey, BMA surveys), regionally or locally (e.g. NHS Trusts). The context of measurement can be at work only, or life in general. It can be measured quantitatively or quantitatively depending on the context. It could be measured in terms of individual wellbeing currently or over a set or undefined time period, and in terms of concepts or determinants.

Pathologies are often used to describe wellbeing; in the systematic review of doctor wellness ‘burnout’ was the most common outcome measured [8]. This is understandable given the lack of a consensus definition and the tradition for pathology to be studied and described in medicine, but hinders progress on what wellbeing is, how it should be measured, and supported.

These factors emphasize the need for a careful analysis and synthesis of findings into a comprehensive summary of how wellbeing measurement in doctors has been undertaken. To provide a knowledge base on which a Delphi consensus can be built to address these issues, a systematic review is normally undertaken to identify possible outcomes and outcome measurement instruments [11] and that approach is undertaken here.

The research questions were: first, ‘which wellbeing outcomes have been measured in doctors?’, and second, ‘which wellbeing outcome measurement instruments have been used with doctors?’.

## METHODS

No existing systematic review answered the research questions, so a protocol to answer them was developed and registered with Prospero (Prospero ID: CRD42020141866). This systematic review followed the Preferred Reporting Items for Systematic Review and Meta-Analysis (PRISMA) checklist [12].

The participant, intervention, comparison, outcome (PICO) method of identifying key concepts was utilized and adapted to the research questions to determine eligibility criteria. Participants: all grades and specialities of doctor. Intervention: for the research questions posed in this review no intervention needed to be captured, but the measurement of wellbeing was the process that needed to be captured. Comparison: no control or comparative measure was required. Outcome: Wellbeing was the outcome of interest.

The definition of wellbeing utilized was ‘Wellbeing is a state of positive feelings and meeting full potential in the world. It can be measured subjectively and objectively, using a salutogenic approach’ [1]. This definition was used as it was important that this review should capture objective as well as subjective, hedonist and eudemonic outcomes. It was also important for the synthesis and discussion of the results that measures could be grouped; into those that measure wellbeing, those that measure pathologies and negative outcomes, and those measuring positive concepts other than wellbeing. No language restrictions were placed. All types of study were included if they measured, or discussed measurement of, doctors’ wellbeing, for any purpose, including reviews and opinion pieces: including qualitative and quantitative measures, and measures which have been recommended but not yet utilized.

The following databases were searched with no restrictions to identify information sources. Bibliographic databases: MEDLINE, EMBASE and Cochrane Central Register of Controlled Trials (CENTRAL). Subject-specific databases: PsycINFO and International Bibliography of Social Science.

The search strategy (Online Supplemental File S1, available as Supplementary data at *Occupational Medicine* Online) was applied with no defined time period or language on 25 November 2019 (the first reported cluster of probable COVID-19 was reported in December 2019).

In the selection process, titles and abstracts were assessed to allow irrelevant reports to be excluded by two researchers independently. When there was a disagreement about eligibility this was arbitrated by a third reviewer. Full-text versions of the potentially relevant reports were sought to assess their eligibility further. Papers that met the criteria had data extracted using a standardized form that allowed the planned outcomes to be captured.

Data were extracted, collated, and assessed in Microsoft Excel [13] using a data extraction form designed prior to extraction. If needed, authors were contacted for any missing data.

The following data items were collected: the context of the studies (study type, year of publication and country conducted in), the sample studied (average age, gender proportions, specialities studied and grades studied), study bias (number approached, number responded and number at follow-up) and the mechanism of measurement of wellbeing (wellbeing in the title, operationalization of wellbeing, how it was described as an outcome [as an aim, in the methods or in the results], delivery method for surveys, outcomes used to capture wellbeing and the measurement instruments used to capture outcomes).

To reduce study risk of bias, results from running the search strategies in the bibliographic databases were exported and merged in Endnote [14]. Duplicate records of the same report were identified using the endnote ‘find duplicates’ function (year, title, volume, issue and pages) and by manual searching and removed. Multiple reports of the same study were linked. Two reviewers screened the studies and used the search function in endnote click [15] to identify where wellbeing was operationalized.

Due to the varied methodology, interventions and outcome measures used, a narrative synthesis was conducted. The operationalization of wellbeing was conceptualized in three categories. First, studies that listed wellbeing as an explicit outcome in the results section. Second, studies that listed wellbeing as an explicit outcome in the methods section (but did not use the word wellbeing in the results). Finally, studies that listed wellbeing as a measurement aim in the introduction or background (with no explicit mention of wellbeing in the methods or results).

Outcomes and outcome measurement instruments were identified using the following criteria: outcomes and measurement instruments that captured wellbeing, as defined by this study and outcomes and measurement tools that did not do so.

There were many outcomes and measurement instruments that did not measure wellbeing but other positive concepts. They were either general or specific to the context of being at work, or being a doctor, and therefore the conceptual model of context was used: general positive concepts, work-specific concepts or doctor-specific concepts.

The outcomes and measurement instruments that were not constructed to capture positive concepts were further categorized into pathologies or symptoms of pathologies. Outcome measurement instruments were categorized as above and as published, unpublished, quantitative or qualitative.

An update search at the end of the review process was not undertaken as this was a snapshot methodological review to capture measurement before the introduction of wellbeing roles within NHS Trusts as recommended by the NHS staff and learners’ mental wellbeing commission and before Covid [9].

The use of this method of categorising the operationalization of a poorly defined concept allowed the reviewers to capture all the ways wellbeing had been measured. This methodology removed the bias of pre-defining what outcomes and/or measurement tools would be included when it was the methodology that was being studied.

To estimate the sensitivity of the search strategy: the percentage of relevant reports found out of the total in existence, the total number of relevant reports identified through database searches was divided by the total number of relevant reports identified through database searches and relevant systematic review backward citation searches. A total of 199 of the 235 relevant studies were identified by the search strategy.

The precision of the search strategy: the percentage of results found that were relevant out of all the results found was calculated by dividing the total number of relevant reports identified through database searches by the total number of irrelevant reports found through both systematic and backward citation searches. It was calculated that 199 of the 4931 studies from the searches were relevant.

The University of Southampton Ethics Committee approved this review as part of a core outcome set development study ERGO55747 and a preprint of the review was published [16].

## RESULTS

**Figure 1.**
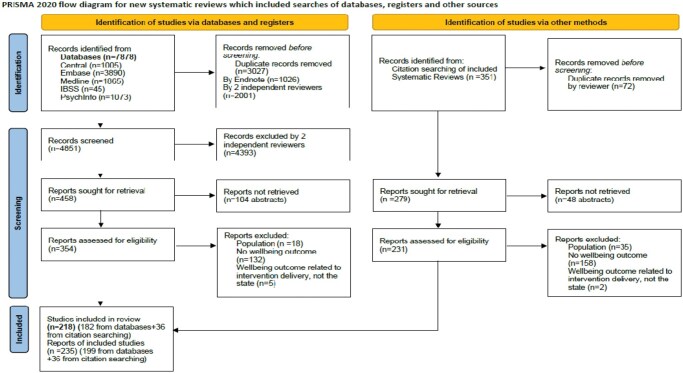
Study selection displayed as a PRISMA flow diagram [252] (CC BY 4.0).

A total of 218 studies were included in this review and are displayed in a PRISMA flow diagram [17] (see Figure 1).

The 218 eligible studies [10,18–239] were heterogeneous in the contexts in which they studied doctors, the outcomes and outcome measurement instruments used. The growth of publications on doctor wellbeing since 1985 was exponential. Studies that measured wellbeing to demonstrate the efficacy of an intervention (see Table 1) comprised 35 studies [18–52], and 157 [53–207] captured the epidemiology of wellbeing in doctors. The USA was the origin of the most studies, 85 [18–20,23–26, 28,30,35, 36, 42, 47, 50, 51, 55, 57, 59, 62, 64, 67, 69, 74, 75, 85, 88, 90, 91, 93, 98, 99, 101, 102, 105, 107–109, 111, 113, 114, 116, 117, 121, 125, 131–133, 136, 139, 145, 147, 150, 152, 156, 159–162, 166, 168, 174, 175, 185–187, 189, 193, 194, 196, 200–203, 207–217] of the 218 studies and only 9 [53, 70, 100, 103, 104, 135, 144, 183, 191] studies were conducted in non-Western cultures. One hundred and six [18, 20, 22, 24, 27, 28, 30, 31, 36, 43–45, 47, 49–51, 53, 55, 56, 58–60, 64–66, 68–71, 76–79, 82, 84–89, 92, 95, 96, 99, 101, 103–108, 110, 115, 117, 121, 126, 135, 139, 141, 143, 144, 146, 147, 156, 162–165, 169, 172, 173, 176, 179, 181–188, 191–193, 195, 197, 198, 203, 206, 207, 209, 210, 213–215, 217–226] studies were undertaken in mixed speciality doctor populations and 134 [10, 23, 24, 26, 28, 29, 33, 35, 39–42, 45, 47, 49, 50, 53, 54, 56–67, 69–72, 76–79, 82, 84, 92, 94, 96, 97, 99–101, 105, 106, 109–111, 115, 117–128, 130, 131, 133–135, 137–141, 143, 144, 149–151, 153, 158, 159, 163, 165, 167, 169, 176, 179–181, 183–185, 188, 191–193, 195, 197–199, 201–205, 207, 210, 211, 214–217, 219, 220, 222–224, 226–234] in mixed grades of doctor. Those studies that captured the age of participants [20, 21, 23, 25–27, 31, 34, 44, 50, 52, 58, 61–64, 66, 70, 71, 73, 74, 76–85, 87, 95, 98, 101, 103, 105, 109, 113, 115, 117, 127, 129, 131, 132, 135, 138, 143–145, 148, 150, 152, 155, 157, 159, 160, 163–166, 168, 170, 172, 174, 181–185, 189, 191–193, 195–197, 199, 201, 202, 206, 211, 213, 219, 224] had a pooled median age of 38.9 years. Collectively, when gender was recorded [18, 20, 21, 23, 25–27, 31, 33–35, 37, 40, 42–45, 47, 52–56, 58, 59, 62–64, 66, 68, 72, 73, 75–78, 80–91, 94–101, 103–109, 112, 113, 115–120, 123, 126–133, 135, 136, 138, 142, 144–152, 154–176, 180–187, 189, 191–200, 202, 203, 206, 211, 213, 219, 221, 223, 224, 226, 234], half of the studies’ populations were male.

**Table 1.** Summary of interventional study findings

Total number of studies *n* = 218	Wellbeing operationalized in results total *n* = 142	Wellbeing operationalized in methods total *n* = 49	Wellbeing operationalized in methods total *n* = 27
Interventional studies *n* = 35	Wellbeing operationalized in results *n* = 16 interventional	Wellbeing operationalized in methods *n* = 16 interventional	Wellbeing operationalized in aims *n* = 3 interventional
Primary interventions *n* = 8	Duty hour policy [18]	Duty hours [19,20]	
	Rest breaks [21]	Rota design [22]	
	Treadmill desks [23]		
	Site improvement plan implementation [24]	Operating Theatre noise [25]	
Secondary interventions *n* = 27	Mindfulness [26–30]	Mindfulness training [31–34]	Mindfulness [35,36]
	Solution focused seminars [37]	Cognitive behavioural therapy [38,39]	Cognitive behavioural therapy [40]
	Dialogue groups [41,42]	Debriefing sessions [43],	
	Peer mentoring [44]	Health and wellbeing workshops [45]	
	Reflection rounds [46]	Meditation training [47,48]	
	Management training [49]	Stress management training [50]	
	Resident assessment facilitation team meetings [51]	Relaxation CDs [52]	

**Table 2.** Summary of findings table, the way in which wellbeing was operationalized and measured. Studies could capture more than one outcome

**Number of**	Operationalization of wellbeing			
	In the aims	In the methods	In the results	Totals
	27 [35,36,40,53–59,96,97, 129, 197, 198, 200–207, 217, 226, 253]	49 [19, 20, 22, 25, 31–34, 38, 39, 43, 45, 47, 48, 50, 52, 66–74, 94, 95, 179, 181–196, 214–216]	142 [18, 21, 24, 27–30, 37, 41, 42, 49, 51, 61–64, 76, 79, 82–89, 92, 93, 101–104, 106, 111, 113–118, 121, 122, 124–126, 129, 131, 133, 134, 138–141, 143, 145–158, 163–165, 169, 170, 172–174, 178, 180, 211–213, 218, 220–224, 227, 234, 254, 255]	218
Studies with wellbeing in the title	9 [56, 58, 59, 96, 97, 197, 201, 202, 204]	17 [22, 39, 45, 48, 66, 68, 71, 179, 187–189, 192, 194, 196, 214, 215]	67 [24, 42, 44, 51, 77, 78, 80–83, 86, 88, 91, 93, 99, 102, 104, 105, 107–109, 114, 116, 119, 121, 124–126, 131–133, 136, 138, 140, 143–147, 151, 152, 155, 156, 158, 162–165, 170, 172, 173, 175, 180, 209–211, 213, 218, 220, 221, 223, 224, 227]	93
General Wellbeing outcomes	10 [35, 36, 40, 53, 58, 59, 96, 205, 206, 217]	30 [19, 20, 22, 25, 31–34, 38, 43, 69, 70, 72–74, 179, 182, 183, 187–189, 191, 195, 196, 214, 215]	116 [18, 21, 24, 27–30, 37, 41, 42, 49, 51, 61–64, 76, 79, 82–89, 92, 93, 101–104, 106, 111, 113–118, 121, 122, 124–126, 129, 131, 133, 134, 138–141, 143, 145–158, 163–165, 169, 170, 172–174, 178, 180, 211–213, 218, 220–224, 227, 234, 254, 255]	156
Positive concept outcomes	12 [55, 58, 60, 96, 197, 198, 201, 204]	9 [47, 50, 52, 183–185, 216]	17 [23, 75, 81, 88, 90, 107–109, 123, 127, 144, 160, 166, 168, 176, 210, 219]	38
Work specific outcomes	28 [53–57, 96, 97, 197–199, 201, 203, 204, 207, 226; 55, 56, 197, 199, 200, 203, 204, 207, 226]	13 [47, 66, 68, 71, 183–185, 193, 216]	40 [49, 75, 77, 80, 109, 123, 127, 135, 137, 162, 172, 175, 176, 210]	81
Symptom of pathology outcomes	10 [54, 55, 96, 198, 199, 201, 202]	16 [22, 39, 45, 48, 66, 68, 94, 181, 186, 190, 194, 216]	20 [49, 77, 78, 81, 98, 107–109, 120, 123, 130, 135, 161, 166, 172, 176, 209, 219]	46
Pathology outcomes	13 [36, 56, 96, 197, 198, 200, 202, 203, 207, 226]	12 [45, 47, 67, 71, 94, 181, 183, 185, 216]	23 [49, 75, 80, 81, 90, 98, 107, 108, 127, 135, 137, 160, 166, 176, 209]	48

The 35 interventional studies [18–52] examined mainly secondary interventions (*n* = 27 studies), that is, group interventions that aim to strengthen the individual, rather than primary interventions that aim to strengthen the wider system (see Table 2). Mindfulness was the most studied secondary intervention, studied in 11 of the interventional studies [26–36].

The majority of eligible studies were cross-sectional surveys (*n* = 112) [98–207] capturing epidemiological data. Of these, 45 [53–97] did so at more than one time-point, and 112 [98–207] at only one time-point, making the majority of studies unsuitable to investigate causality, and at risk of sampling bias and conformity bias. This would have been mitigated by 155 studies [75, 98–100, 102, 103, 105, 106, 109, 111–113, 115–120, 122–124, 126, 127, 131, 135–140, 142–152, 154–157, 159–167, 169–174, 176, 178–182, 184–190, 192–199, 202–207, 226] being conducted in multiple centres; however, most multi-centre studies were undertaken in hospitals in the USA, through the same healthcare provider group [98, 99, 102, 105, 109, 111, 113, 116, 117, 131, 136, 139, 145–147, 150, 152, 156, 159–162, 166, 174, 185–187, 189, 193, 194, 196, 202, 203, 207, 211]. In terms of selection bias, response rates for the cross-sectional surveys could be established in 76 [98, 99, 102–109, 112–117, 119–125, 148, 150–154, 156, 157, 159, 160, 162, 164, 166, 168, 170, 171,174–176,178–183, 185–187, 190, 191, 193, 195, 196, 199–207, 211, 226] of 112 studies [98–207]: the median percentage response rate was 55.2 (range 0.9–99). Of the 35 [18–52] studies that were interventional, 22 used a control [18, 19, 21–23, 26–29, 31–33, 38, 40, 42–45, 47, 49, 51, 52] and 15 used randomization [18, 19, 21, 22, 26, 27, 31–33, 42–45, 47, 52].

Regarding performance bias, all the interventional studies were unable to ‘blind’ participants, as doctors would be aware of what all the interventions comprised.

Given the knowledge of doctors and their professional status there is potential for them to answer self-report outcomes more ‘correctly’ than the general population, to conform with social desirability. This detection bias would have been more problematic in the non-anonymous outcome collection methods.

For the prospective observational studies, cohort studies, randomized controlled trials, non-randomized controlled studies and interventional studies with no control, the percentage of participants lost to follow up (attrition bias) could be calculated in 49 of 80 studies [18–52]: the median percentage attrition rate was 33 (range 0–98).

Where results were missing, the authors were not contacted as this methodological review aimed to describe what was published about study design. Some interpretation was required to identify which measurement tool was chosen to capture the outcome wellbeing, usually involving a process of elimination as other outcome concepts were better defined and their measurement instruments could be paired with them more easily. The systematic reviews included did not describe their identification process [10, 228, 230, 231, 233,235–239].

Synthesis of the results to answer research question 1: the outcomes measure in doctors showed the total number of unique outcomes used to capture wellbeing in all the eligible studies was 57. Unique outcomes were those that represented a novel concept. Non-unique outcomes were those that described the same concept, using different wording. The total count of non-unique wellbeing outcomes used in the 218 studies was 369. One hundred and fifty-six of the 369 non-unique outcomes contained the word ‘wellbeing’. One hundred and ninety-four of the non-unique outcomes contained the word wellbeing, or its components or other positive concepts. If the positive work-context outcomes were also counted 258 positive outcomes were used. One hundred and eleven negative concept outcomes were used such as negative work context outcomes, symptoms of pathologies, or pathologies.

Synthesis of the results for research question 2: the outcome measurement instrument used showed that for the outcome ‘general wellbeing’ 92 different measurement tools were used, the most commonly employed being shown in Table 3. The measurement tools used could be classed as published wellbeing measurement tools (*n* = 9), published measurement tools for positive concepts other than wellbeing (*n* = 13), doctor-specific wellbeing measurement tools (*n* = 10), work-specific measurement tools (*n* = 9), measurement tools for symptoms of pathologies (*n* = 6), pathology screening tools (*n* = 13), unpublished study-specific measurement tools (*n* = 20) and qualitative tools (*n* = 12).

**Table 3.** The most commonly used published outcome measurement instruments to capture the outcome general wellbeing

**Measurement tool**	Description of tool	Number of times used	References
Maslach Burnout Inventory	Published pathology screening tool	8	[18, 33, 148, 150, 206, 214, 215, 218]
WHO wellbeing index (5 item 5-point)	Published wellbeing measurement tool	5	[27, 29, 83, 126, 218]
One item VAS (100 mm)	Published wellbeing measurement tool	5	[61, 63, 93, 129, 148]
LASA QOL	Published positive concept measurement tools	4	[99, 125, 131, 151, 211]
GHQ12	Published pathology screening tools	4	[38, 143, 165, 169]
Dupuy Psychological General Wellbeing scale	Published wellbeing measurement tool	3	[65, 145–147]
Physician wellbeing index	Published ­doctor-specific measurement tools	3	[28, 117, 215]
Stanford professional fulfilment (PFI)	Published ­doctor-specific measurement tools	3	[36, 215, 217]

WHO (World Health Organisation), VAS (Visual Analogue Scale), LASA (Linear Analog Scale Assessment), QOL (Quality of Life), GHQ (General Health Questionnaire)

The Maslach Burnout Inventory [240] was the most frequently used measurement tool for all intended wellbeing outcomes, used in 34 of the studies [22, 33, 37, 47, 71, 81, 90, 98, 107, 108, 110, 116, 127, 137, 148, 150, 160, 166, 171, 175, 181, 195, 202, 205, 206, 209, 214, 215, 218, 231, 233, 238]; 22 studies that operationalized wellbeing as an explicit outcome in the results section [37,81,90,98,107,108,110,116,127,137,148,150,160,166,171,175,209,218,231,233], nine studies that operationalized wellbeing as an explicit outcome in the methods [22, 33, 47, 71, 181, 195, 214, 215, 238] and three studies that stated wellbeing measurement was an explicit aim [202, 205, 206].

## DISCUSSION

The principle findings of this study were that first the word ‘wellbeing’ was used in the title of 93 studies, and more so in studies that operationalized wellbeing in the results (*n* = 67). Second, each of the different ways of operationalising wellbeing included in this review (as an aim, or as an outcome in the methods, or in the results) was justified as each approach identified 10, 6 and 12 unique outcomes, respectively. Third, in a systematic review of physician wellness [10] 24 pre-defined ‘dimensions’ of wellness were used to categorise 75 non-unique outcomes in 43 studies published between 2010 and 2015. In comparison, this review identified 57 unique wellbeing outcomes and 369 non-unique outcomes. Including studies that operationalized wellbeing as a measurement aim, or as an outcome in the methods, as well as in the results, allowed identification of all the ways that wellbeing in doctors has been measured. The heterogeneity of how doctor wellbeing was operationalized highlights both its multi-faceted nature and the need for a consensus approach to wellbeing research in doctors. A lack of operational definitions is not a problem unique to reviews of wellbeing research. A systematic review of core outcome set development studies found that no study defined how outcomes were differentiated and how final numbers of unique outcomes were determined [241].

The 92 measurement tools used to capture the outcome ‘general wellbeing’ alone again reflects the lack of a consensus operational definition for wellbeing, as does the finding that 17 of these measurement tools were self-created. The finding that the Maslach Burnout Inventory, despite its associated cost, was the most commonly used tool shows a desire for a well-operationalized concept and psychometrically tested tool. The use of a pathological outcome measurement instrument is worrying, as it limits the best a doctor can achieve to a lack of mental ill health. The finding that 30.1% of outcomes used to capture wellbeing were pathological or negative concepts reflects the ‘medicalization’ of the concept of wellbeing, which is inherently positive and holistic.

A strength of this systematic review was that it included a large number of studies (*n* = 218) compared to the previous systematic reviews identified through database searches [10, 242–249], which identified a median of 28.5 eligible studies (range 3–81). The inclusion of all study types and languages, as well as searching diverse databases should have reduced selection bias in this systematic review. Reporting bias will be present, as published work is accessed more easily and it was not practically possible to search ‘grey literature’, given the 7878 studies retrieved in the existing database searches.

This systematic review utilized a novel way of categorising how a poorly defined concept—‘wellbeing’—is conceptualized in publications. It achieved this by coding whether the concept featured in the aims, method or results. This strategy enabled Adobe Acrobat Pro DC [250] and Endnote click [15] to be used to identify where the concept was mentioned, which reduced reviewer error and subjective bias as reviewers are only required to identify if the word is being used as an outcome.

The inductive collection of outcomes without a pre-conceived framework used in this systematic review has not been undertaken in other systematic reviews of wellbeing measurement [2, 3, 10, 251]; providing a more complete picture of the doctor wellbeing landscape by allowing for a comprehensive synthesis of all methods for measuring doctor wellbeing.

To mitigate selection bias, double screening was used with 10% of full-text articles being double-screened at the ‘title and abstract’ stage. Reviewers were not blind to authors and institutions, but these data did not need to be read to assess for eligibility; selection bias risk was therefore low. Ideally, all studies would have had data extracted by two independent researchers, but this was only possible for 86 of the reports. An audit was performed of 10% of the extracted outcome data for each of the three ways wellbeing could be operationalized: as an aim, in the methods or in the results, with no disagreements. It was not possible to comment on the sensitivity of this review compared to others, as the only other methodological systematic review in this area did not report the necessary data [10]. Future work could compare published methodology with unpublished methodology through interviews with authors to further capture the operationalization of outcomes, the choice of measurement instrument and the impact of this on reporting bias.

The heterogeneity of outcomes used to measure doctor wellbeing hinders comparisons between studies looking at epidemiology or the efficacy of interventions and therefore hinders policy makers in supporting clinicians. There is no consensus about which measurement tools are appropriate for the measurement of doctor wellbeing, leading to many different and often self-created tools being utilized. Meta-analysis will not be possible until the same outcome measurement instruments start to be used.

Wellbeing has been measured heterogeneously in doctors in terms of the outcomes and the outcome measurement instruments used. Just under a third of the times it was measured, the best that could be achieved was an absence of pathological symptoms, as a negative concept was used to operationalize it.

The results of this systematic review highlight the importance of core outcome sets, in which a minimum agreed set of outcomes and recommended measurement tools to capture them allow comparisons across studies. The use of a core outcome set, including recommended outcome measurement instruments would provide the optimal environment for synthesis and meta-analysis to occur in the field of staff wellbeing.

## Supplementary Material

kqaf088_Supplementary_Data

## Data Availability

All data supporting this study are openly available from the University of Southampton repository at http://dx.doi.org/10.5258/SOTON/D2318.

## TRIAL REGISTRY

Prospero ID: CRD42020141866.
