# Supplementary material for: Measurement of doctor wellbeing prior to the Covid pandemic: a methodological systematic review
Source: Occup Med (Lond). 2025 Oct 6;75(9):612–9. doi: 10.1093/occmed/kqaf088 (PMC12794918; doi:10.1093/occmed/kqaf088)
Supplement: kqaf088_Supplementary_Data [file kqaf088_supplementary_data.docx]

## Supplementary File 3 Complete Reference List

71. Dowie R, Stoykova B, Desai M. Assessing the wellbeing of cytoscreeners: experience in two NHS cytology laboratories. Cytopathology : official journal of the British Society for Clinical Cytology 2006;17(6):366-73. doi: https://doi.org/10.1111/j.1365-2303.2006.00383.x.

72. Downey GB, McDonald J, Downey RG. Welfare of anaesthesia trainees survey. Anaesthesia and Intensive Care 2017;45(1):73-78. doi: https://doi.org/10.1177/0310057x1704500111.

73. Zahrai A, Bhandari M, Varma A, et al. Residents' quality of life during an orthopedic trauma rotation: a multicentre prospective observational study. Can J Surg 2008;51(3):190-6. Available from: https://www.ncbi.nlm.nih.gov/pubmed/18682757. (accessed 17/01/2022).

74. Eisenach JH, Sprung J, Clark MM, et al. The psychological and physiological effects of acute occupational stress in new anesthesiology residents: a pilot trial. Anesthesiology 2014;121(4):878-93. doi: https://doi.org/10.1097/aln.0000000000000397.

75. Antiel RM, Reed DA, Van Arendonk KJ, et al. Effects of duty hour restrictions on core competencies, education, quality of life, and burnout among general surgery interns. JAMA surgery 2013;148(5):448-55. doi: http://dx.doi.org/10.1001/jamasurg.2013.1368.

76. Buddeberg-Fischer B, Klaghofer R, Stamm M, et al. Work stress and reduced health in young physicians: prospective evidence from Swiss residents. International archives of occupational and environmental health 2008;82(1):31-8. doi: https://dx.doi.org/10.1007/s00420-008-0303-7.

77. Elovainio M, Heponiemi T, Jokela M, et al. Stressful work environment and wellbeing: What comes first? Journal of Occupational Health Psychology 2015;20(3):289-300. doi: http://dx.doi.org/10.1037/a0038684.

78. Elovainio M, Salo P, Jokela M, et al. Psychosocial factors and well-being among Finnish GPs and specialists: a 10-year follow-up. Occupational and environmental medicine 2013;70(4):246-51. doi: https://dx.doi.org/10.1136/oemed-2012-100996.

79. Finset KB, Gude T, Hem E, et al. Which young physicians are satisfied with their work? A prospective nationwide study in Norway. BMC medical education 2005;5(1):19. doi: https://doi.org/10.1186/1472-6920-5-19.

80. Mason S, O'Keeffe C, Carter A, et al. A longitudinal study of well-being, confidence and competence in junior doctors and the impact of emergency medicine placements. Emergency medicine journal : EMJ 2016;33(2):91-8. doi: https://dx.doi.org/10.1136/emermed-2014-204514.

81. Meier LL, Tschudi P, Meier CA, et al. When general practitioners don't feel appreciated by their patients: prospective effects on well-being and work-family conflict in a Swiss longitudinal study. Family practice 2015;32(2):181-6. doi: https://dx.doi.org/10.1093/fampra/cmu079.

82. Mullola S, Hakulinen C, Gimeno Ruiz de Porras D, et al. Medical specialty choice and well-being at work: Physician's personality as a moderator. Archives of Environmental and Occupational Health 2019;74(3):115-29. doi: http://dx.doi.org/10.1080/19338244.2018.1448355.

83. Noroxe KB, Pedersen AF, Carlsen AH, et al. Mental well-being, job satisfaction and self-rated workability in general practitioners and hospitalisations for ambulatory care sensitive conditions among listed patients: A cohort study combining survey data on GPs and register data on patients. BMJ Quality and Safety 2019. doi: http://dx.doi.org/10.1136/bmjqs-2018-009039.

84. Rosta J, Aasland OG. Changes in the lifetime prevalence of suicidal feelings and thoughts among Norwegian doctors from 2000 to 2010: A longitudinal study based on national samples. BMC Psychiatry 2013;13. doi: https://doi.org/10.1186/1471-244X-13-322.

85. Sen S, Kranzler HR, Didwania AK, et al. Effects of the 2011 duty hour reforms on interns and their patients: A prospective longitudinal cohort study. JAMA Internal Medicine 2013;173(8):657-62. doi: http://dx.doi.org/10.1001/jamainternmed.2013.351.

86. Smith F, Goldacre MJ, Lambert TW. Adverse effects on health and wellbeing of working as a doctor: views of the UK medical graduates of 1974 and 1977 surveyed in 2014. Journal of the Royal Society of Medicine 2017;110(5):198-207. doi: http://dx.doi.org/10.1177/0141076817697489.

87. Tartas M, Walkiewicz M, Budzinski W, et al. The sense of coherence and styles of success in the medical career: a longitudinal study. BMC medical education 2014;14:254. doi: http://dx.doi.org/10.1186/s12909-014-0254-5.

88. West CP, Shanafelt TD, Cook DA. Lack of association between resident doctors' well-being and medical knowledge. Medical Education, 2010;44(12):1224-31. doi: https://dx.doi.org/10.1111/j.1365-2923.2010.03803.x.

89. Zazulak J, Sanaee M, Frolic A, et al. The art of medicine: arts-based training in observation and mindfulness for fostering the empathic response in medical residents. Medical humanities 2017;43(3):192-98. doi: http://dx.doi.org/10.1136/medhum-2016-011180.

90. West CP, Shanafelt TD, Kolars JC. Quality of life, burnout, educational debt, and medical knowledge among internal medicine residents. JAMA: Journal of the American Medical Association 2011;306(9):952-60. doi: https://doi.org/10.1001/jama.2011.1247.

91. Salles A, Cohen GL, Mueller CM. The relationship between grit and resident well-being. American Journal of Surgery, 2014;207(2):251-4. doi: https://doi.org/10.1016/j.amjsurg.2013.09.006.

92. Meerten M, Bland J, Gross SR, et al. Doctors' experience of a bespoke physician consultation service: cross-sectional investigation. The Psychiatrist 2011;35(6):206-12. doi: http://dx.doi.org/10.1192/pb.bp.110.031047.

93. Stamp T, Termuhlen P, Miller S, et al. Before and after resident work hour limitations: an objective assessment of the well-being of surgical residents. Current Surgery, 2005;62(1):117-21. doi: https://doi.org/10.1016/j.cursur.2004.09.013.

94. Jones N, Whybrow D, Coetzee R. UK military doctors; stigma, mental health and help-seeking: a comparative cohort study. BMJ Military Health, 2018;164(4):259-66. doi: http://dx.doi.org/10.1136/jramc-2018-000928.

95. Tyssen R, Hem E, Gude T, et al. Lower life satisfaction in physicians compared with a general population sample: A 10-year longitudinal, nationwide study of course and predictors. Social Psychiatry and Psychiatric Epidemiology: The International Journal for Research in Social and Genetic Epidemiology and Mental Health Services 2009;44(1):47-54. doi: https://doi.org/10.1007/s00127-008-0403-4.

96. Cvejic E, Parker G, Harvey SB, et al. The health and well-being of Australia's future medical doctors: Protocol for a 5-year observational cohort study of medical trainees. BMJ Open 2017;7(9):e016837. doi: http://dx.doi.org/10.1136/bmjopen-2017-016837.

97. Eley DS, Robert Cloninger C, Walters L, et al. The relationship between resilience and personality traits in doctors: Implications for enhancing well being. PeerJ 2013;2013(1):216. doi: http://dx.doi.org/10.7717/peerj.216.

98. Sun H, Warner DO, Macario A, et al. Repeated Cross-sectional Surveys of Burnout, Distress, and Depression among Anesthesiology Residents and First-year Graduates. Anesthesiology 2019;131(3):668-77. doi: https://doi.org/10.1097/ALN.0000000000002777.

99. Tawfik DS, Profit J, Morgenthaler TI, et al. Physician Burnout, Well-being, and Work Unit Safety Grades in Relationship to Reported Medical Errors. Mayo Clinic Proceedings 2018;93(11):1571-80. http://dx.doi.org/10.1016/j.mayocp.2018.05.014. doi: http://dx.doi.org/10.1016/j.mayocp.2018.05.014.

100. Hamdan M, Abu Hamra A. Workplace violence towards workers in the emergency departments of Palestinian hospitals: a cross-sectional study. Human resources for health 2015;13:28. doi: http://dx.doi.org/10.1186/s12960-015-0018-2.

101. Gates R, Musick D, Greenawald M, et al. Evaluating the Burnout-Thriving Index in a Multidisciplinary Cohort at a Large Academic Medical Center. Southern Medical Journal 2019;112(4):199-204. doi: http://dx.doi.org/10.14423/SMJ.0000000000000962.

102. Adams PS, Gordon EKB, Berkeley A, et al. Academic faculty demonstrate higher well-being than residents: Pennsylvania anesthesiology programs' results of the 2017-2018 ACGME well-being survey. Journal of Clinical Anesthesia 2019;56:60-64. doi: http://dx.doi.org/10.1016/j.jclinane.2019.01.037.

103. Al Huseini S, Al Alawi M, Al Sinawi H, et al. Trait Emotional Intelligence and Its Correlates in Oman Medical Specialty Board Residents. Journal of graduate medical education 2019;11(4 Supplement):134-40. doi: http://dx.doi.org/10.4300/JGME-D-18-00388.

104. Alatiq Y. Stress, depressive symptoms, well-being and mindfulness in sample of Saudi medical residents. Arab Journal of Psychiatry 2016;27(2):144-50. Available from: https://www.researchgate.net/publication/321005127_Stress_depressive_symptoms_well-being_and_mindfulness_in_sample_of_Saudi_medical_residents. (accessed 17/01/2022).

105. Albanese-Kotar NF. Development of an expanding typology of perfectionism and examination of the relationship between perfectionism and psychological well-being. ProQuest Information & Learning, 2001.

106. Arnetz BB. Staff perception of the impact of health care transformation on quality of care. International journal for quality in health care 1999;11(4):345-51. doi: https://doi.org/10.1093/intqhc/11.4.345.

107. Beckman TJ, Reed DA, Shanafelt TD, et al. Impact of resident well-being and empathy on assessments of faculty physicians. Journal of general internal medicine 2010;25(1):52-6. doi: https://dx.doi.org/10.1007/s11606-009-1152-0.

108. Beckman TJ, Reed DA, Shanafelt TD, et al. Resident physician well-being and assessments of their knowledge and clinical performance. Journal of general internal medicine 2012;27(3):325-30. doi: https://dx.doi.org/10.1007/s11606-011-1891-6.

109. Busis NA, Shanafelt TD, Keran CM, et al. Burnout, career satisfaction, and well-being among US neurologists in 2016. Neurology 2017;88(8):797-808. doi: http://dx.doi.org/10.1212/WNL.0000000000003640.

110. Catt S, Fallowfield L, Jenkins V, et al. The informational roles and psychological health of members of 10 oncology multidisciplinary teams in the UK. British journal of cancer 2005;93(10):1092-7. doi: https://doi.org/10.1038/sj.bjc.6602816.

111. Cohen-Katz J, Sternlieb JL, Hansen SE, et al. Developing Emotional Intelligence in the Clinical Learning Environment: A Case Study in Cultural Transformation. Journal of graduate medical education 2016;8(5):692-98. doi: http://dx.doi.org/10.4300/JGME-D-15-00548.1.

112. Cooper CL, Rout U, Faragher B. Mental health, job satisfaction, and job stress among general practitioners. BMJ (Clinical research ed.) 1989;298(6670):366-70. doi: https://dx.doi.org/10.1136%2Fbmj.298.6670.366.

113. Cull WL, Katakam SK, Starmer AJ, et al. A Study of Pediatricians' Debt Repayment a Decade After Completing Residency. Academic medicine, 2017;92(11):1595-600. doi: http://dx.doi.org/10.1097/ACM.0000000000001721.

114. Danhakl V, Miltiades A, Ing C, et al. Observational study evaluating obstetric anesthesiologist residents' well-being, anxiety and stress in a North American academic program. International Journal of Obstetric Anesthesia 2019;38:75-82. doi: http://dx.doi.org/10.1016/j.ijoa.2018.10.011.

115. Davies SR, Meerton M, Rost F, et al. A sea change for sick doctors - how do doctors fare after presenting to a specialist psychotherapy service? Journal of mental health (Abingdon, England) 2016;25(3):238-44. doi: https://dx.doi.org/10.3109/09638237.2015.1124386.

116. Dodson KM, Appelbaum NP, Lee N, et al. Otolaryngology Resident Well-Being and Perceptions of the Clinical Learning Environment. Ear, Nose and Throat Journal 2019;98(7):409-15. doi: http://dx.doi.org/10.1177/0145561319840125.

117. Dyrbye LN, Satele D, Sloan J, et al. Utility of a brief screening tool to identify physicians in distress. Journal of general internal medicine 2013;28(3):421-7. doi: https://dx.doi.org/10.1007/s11606-012-2252-9.

118. Egerton-Warburton D, Gosbell A, Wadsworth A, et al. Perceptions of Australasian emergency department staff of the impact of alcohol-related presentations. Medical Journal of Australia 2016;204(4):155. doi: http://dx.doi.org/10.5694/mja15.00858.

119. Escribà-Agüir V, Pérez-Hoyos S. Psychological well-being and psychosocial work environment characteristics among emergency medical and nursing staff. Stress and Health: Journal of the International Society for the Investigation of Stress 2007;23(3):153-60. doi: http://dx.doi.org/10.1002/smi.1131.

120. Evans HJ. A model of resilience, burnout and intention to quit among general practitioners. ProQuest Information & Learning, 2018.

121. Frazier KN. The effect of leadership styles on workplace relationships and employee well-being of medical staff employees. ProQuest Information & Learning, 2014.

122. Gander PH, Merry A, Millar MM, et al. Hours of work and fatigue-related error: A survey of New Zealand anaesthetists. Anaesthesia and Intensive Care 2000;28(2):178-83. doi: https://doi.org/10.1177/0310057x0002800209.

123. Gardiner M, Sexton R, Kearns H, et al. Impact of support initiatives on retaining rural general practitioners. Australian Journal of Rural Health 2006;14(5):196-201. doi: http://dx.doi.org/10.1111/j.1440-1584.2006.00808.x.

124. Grieve S. Measuring morale - Does practice area deprivation affect doctors' well-being? British Journal of General Practice 1997;47(422):547-52. Available from: https://www.ncbi.nlm.nih.gov/pubmed/9406487. (accessed 17/01/2022).

125. Guest RS, Baser R, Li Y, et al. Cancer surgeons' distress and well-being, I: the tension between a culture of productivity and the need for self-care. Annals of surgical oncology 2011;18(5):1229-35. doi: https://dx.doi.org/10.1245/s10434-011-1622-6.

126. Hayes B, Prihodova L, Walsh G, et al. What's up doc? A national cross-sectional study of psychological wellbeing of hospital doctors in Ireland. BMJ Open 2017;7(10):e018023. doi: http://dx.doi.org/10.1136/bmjopen-2017-018023.

127. Johnson S, Osborn DPJ, Araya R, et al. Morale in the English mental health workforce: questionnaire survey. The British journal of psychiatry : the journal of mental science 2012;201(3):239-46. doi: https://dx.doi.org/10.1192/bjp.bp.111.098970.

128. Kinzl JF, Traweger C, Trefalt E, et al. Work stress and gender-dependent coping strategies in anesthesiologists at a university hospital. Journal of Clinical Anesthesia 2007;19(5):334-8. doi: https://doi.org/10.1016/j.jclinane.2006.08.014.

129. Klein M, Andersen LPH, Alamili M, et al. Psychological and physical stress in surgeons operating in a standard or modern operating room. Surgical laparoscopy, endoscopy & percutaneous techniques 2010;20(4):237-42. doi: https://dx.doi.org/10.1097/SLE.0b013e3181ed851d.

130. Klingberg K, Gadelhak K, Jegerlehner SN, et al. Bad manners in the Emergency Department: Incivility among doctors. PLOS ONE 2018;13(3). doi: https://dx.doi.org/10.1371%2Fjournal.pone.0194933.

131. Levin KH, Shanafelt TD, Keran CM, et al. Burnout, career satisfaction, and well-being among US neurology residents and fellows in 2016. Neurology 2017;89(5):492-501. doi: http://dx.doi.org/10.1212/WNL.0000000000004135.

132. Lin DT, Liebert CA, Tran J, et al. Emotional Intelligence as a Predictor of Resident Well-Being. Journal of the American College of Surgeons 2016;223(2):352-58. doi: http://dx.doi.org/10.1016/j.jamcollsurg.2016.04.044.

133. Love AM. Rural clinicians' perceived ethical dilemmas: Relationships with clinician well-being and burnout. ProQuest Information & Learning, 2015.

134. Martins S, Johnston G. Impact of orthopedic trauma consolidation on resident education. Canadian journal of surgery. Journal canadien de chirurgie 2009;52(6):495-9. Available from: https://www.ncbi.nlm.nih.gov/pubmed/20011186. (accessed 17/01/2022).

135. Meir EI, Melamed S, Dinur C. The benefits of congruence. The Career Development Quarterly 1995;43(3):257-66. doi: https://psycnet.apa.org/doi/10.1002/j.2161-0045.1995.tb00866.x.

136. Milam LA, Cohen GL, Mueller C, et al. The Relationship Between Self-Efficacy and Well-Being Among Surgical Residents. Journal of Surgical Education 2019;76(2):321-28. doi: http://dx.doi.org/10.1016/j.jsurg.2018.07.028.

137. Minvielle E, Dervaux B, Retbi A, et al. Culture, organization, and management in intensive care: construction and validation of a multidimensional questionnaire. Journal of critical care 2005;20(2):126-38. doi: https://doi.org/10.1016/j.jcrc.2004.12.003.

138. Murfett A, Charman D. GP wellbeing and general practice issues. Australian family physician 2006;35(9):748-50. Available from: https://www.racgp.org.au/afp/200609/11038. (accessed 17/01/2022).

139. Parsons KG. Moral and ethical decision making of physicians and attorneys: The influence of faith across their career lifespans. ProQuest Information & Learning, 2000.

140. Poulsen MG, Poulsen AA, Khan A, et al. Factors associated with subjective well-being in cancer workers in Queensland. Journal of Medical Imaging and Radiation Oncology 2012;56(3):347-53. doi: http://dx.doi.org/10.1111/j.1754-9485.2012.02368.x.

141. Reid NG, Moss PJ. The impact of the new deal: Doctors' stress levels and their views. Stress Medicine 1999;15(1):9-15. doi: http://dx.doi.org/10.1002/%28SICI%291099-1700%28199901%2915:1%3C9::AID-SMI780%3E3.0.CO;2-O.

142. Rout U. Job stress among British general practitioners: Predictors of job dissatisfaction and mental ill-health. Stress Medicine 1996;12(3):155-66. doi: http://dx.doi.org/10.1002/%28SICI%291099-1700%28199607%2912:3%3C155::AID-SMI687%3E3.0.CO;2-A.

143. Russo A, De Luca R, Cicero G, et al. Well-being among Italian medical oncologists: an exploratory study. Oncology 2014;86(2):72-8. doi: https://dx.doi.org/10.1159/000354642.

144. Sahoo FM, Mohapatra L. Psychological well-being in professional groups. Journal of the Indian Academy of Applied Psychology 2009;35(2):211-17.

145. Salles A, Wright RC, Milam L, et al. Social Belonging as a Predictor of Surgical Resident Well-being and Attrition. Journal of Surgical Education 2019;76(2):370-77. doi: http://dx.doi.org/10.1016/j.jsurg.2018.08.022.

146. Salles A, Milam L, Cohen G, et al. The relationship between perceived gender judgment and well-being among surgical residents. American Journal of Surgery 2018;215(2):233-37. doi: http://dx.doi.org/10.1016/j.amjsurg.2017.08.049.

147. Salles A, Mueller CM, Cohen GL. Exploring the Relationship Between Stereotype Perception and Residents' Well-Being. Journal of the American College of Surgeons 2016;222(1):52-8. doi: https://dx.doi.org/10.1016/j.jamcollsurg.2015.10.004.

148. Schaufeli WB, Bakker AB, van der Heijden FMMA, et al. Workaholism among medical residents: It is the combination of working excessively and compulsively that counts. International Journal of Stress Management 2009;16(4):249-72. doi: https://psycnet.apa.org/doi/10.1037/a0017537.

149. Schooley B, Hikmet N, Tarcan M, et al. Comparing Burnout Across Emergency Physicians, Nurses, Technicians, and Health Information Technicians Working for the Same Organization. Medicine 2016;95(10):e2856. doi: https://dx.doi.org/10.1097/MD.0000000000002856.

150. Shanafelt TD, Gradishar W, Kosty MP, et al. Burnout and career satisfaction among U.S. oncologists: Results of the 2012 ASCO survey. Journal of Clinical Oncology 2013;31(15 SUPPL. 1). doi: https://doi.org/10.1200/jco.2013.51.8480.

151. Shanafelt TD, Novotny P, Johnson ME, et al. The well-being and personal wellness promotion strategies of medical oncologists in the North Central Cancer Treatment Group. Oncology 2005;68(1):23-32. doi: http://dx.doi.org/10.1159/000084519.

152. Shanafelt TD, Raymond M, Horn L, et al. Oncology fellows' career plans, expectations, and well-being: do fellows know what they are getting into? Journal of Clinical Oncology 2014;32(27):2991-7. doi: https://doi.org/10.1200/jco.2014.56.2827.

153. Spear J. Why do health professionals work in a community mental health service? Australasian psychiatry : bulletin of Royal Australian and New Zealand College of Psychiatrists 2006;14(2):175-9. doi: https://doi.org/10.1080%2Fj.1440-1665.2006.02260.x.

154. Stafford L, Judd F. Mental health and occupational wellbeing of Australian gynaecologic oncologists. Gynecologic oncology 2010;116(3):526-32. doi: https://dx.doi.org/10.1016/j.ygyno.2009.10.080.

155. Szafran O, Woloschuk W, Torti JMI, et al. Well-being of family medicine graduates. Canadian family physician Medecin de famille canadien 2017;63(10):e432-e39. Available from: http://www.ncbi.nlm.nih.gov/pmc/articles/pmc5638491/. (accessed 17/01/2022).

156. Tak HJ, Curlin FA, Yoon JD. Association of Intrinsic Motivating Factors and Markers of Physician Well-Being: A National Physician Survey. Journal of general internal medicine 2017;32(7):739-46. doi: http://dx.doi.org/10.1007/s11606-017-3997-y.

157. Taylor DM, Pallant JF, Crook HD, et al. The psychological health of emergency physicians in Australasia. Emergency medicine Australasia : EMA 2004;16(1):21-7. doi: https://doi.org/10.1111/j.1742-6723.2004.00532.x.

158. Wallace JE, Lemaire J. On physician well being--You'll get by with a little help from your friends. Social Science & Medicine 2007;64(12):2565-77. doi: https://doi.org/10.1016/j.socscimed.2007.03.016.

159. Weiner EL, Swain GR, Wolf B, et al. A qualitative study of physicians' own wellness-promotion practices. Western Journal of Medicine 2001;174(1):19-23. doi: http://dx.doi.org/10.1136/ewjm.174.1.19.

160. West CP, Halvorsen AJ, Swenson SL, et al. Burnout and distress among internal medicine program directors: Results of a national survey. Journal of general internal medicine 2013;28(8):1056-63. doi: http://dx.doi.org/10.1007/s11606-013-2349-9.

161. Wiens KJ. Leading through burnout: The influence of emotional intelligence on the ability of executive level physician leaders to cope with occupational stress and burnout. ProQuest Information & Learning, 2017.

162. Yoon JD, Daley BM, Curlin FA. The association between a sense of calling and physician well-being: A national study of primary care physicians and psychiatrists. Academic Psychiatry 2017;41(2):167-73. doi: https://doi.org/10.1007/s40596-016-0487-1.

163. Acuna J, Schiattino I, Horwitz N, et al. Characteristics of employment and wellbeing among physicians working in Santiago, Chile. Caracteristicas del empleo y bienestar subjetivo en medicos de la Region Metropolitana. 2013;141(2):187-93. doi: https://dx.doi.org/10.4067/S0034-98872013000200007.

164. Cohen JS, Patten S. Well-being in residency training: a survey examining resident physician satisfaction both within and outside of residency training and mental health in Alberta. BMC Med Educ 2005;5:21. doi: https://doi.org/10.1186/1472-6920-5-21.

165. Heijstra TM, Rafnsdóttir GL, Jónsdóttir LS. Autonomy and well-being among Nordic male and female hospital physicians. Work 2011;40(4):437-43. doi: https://doi.org/10.3233/wor-2011-1255.

166. Lebensohn P, Dodds S, Benn R, et al. Resident wellness behaviors: relationship to stress, depression, and burnout. Family Medicine 2013;45(8):541-9. Available from: https://www.researchgate.net/publication/257839292_Resident_Wellness_Behaviors_Relationship_to_Stress_Depression_and_Burnout. (accessed 17/01/2022).

167. Lederer W, Kinzl JF, Trefalt E, et al. Significance of working conditions on burnout in anesthetists. Acta Anaesthesiol Scand 2006;50(1):58-63. doi: https://doi.org/10.1111/j.1399-6576.2005.00867.x.

168. Lin GA, Beck DC, Stewart AL, et al. Resident perceptions of the impact of work hour limitations. J Gen Intern Med 2007;22(7):969-75. doi: https://doi.org/10.1007/s11606-007-0223-3.

169. Løvseth LT, Aasland OG, Fridner A, et al. Confidentiality and physicians' health. A cross-sectional study of University Hospital Physicians in four European cities (the HOUPE-study). J Occup Health 2010;52(5):263-71. doi: https://doi.org/10.1539/joh.L10014.

170. Uncu Y, Bayram N, Bilgel N. Job related affective well-being among primary health care physicians. European Journal Public Health 2007;17(5):514-9. doi: https://doi.org/10.1093/EURPUB%2FCKL264.

171. van Vendeloo SN, Brand PL, Verheyen CC. Burnout and quality of life among orthopaedic trainees in a modern educational programme: importance of the learning climate. Bone and Joint Journal 2014;96-b(8):1133-8. doi: https://doi.org/10.1302/0301-620x.96b8.33609.

172. von Vultée PJ, Axelsson R, Arnetz B. The impact of organisational settings on physician wellbeing. International Journal Health Care Quality Assurance 2007;20(6):506-15. doi: https://doi.org/10.1108/09526860710819440.

173. Cohen JS, Leung Y, Fahey M, et al. The happy docs study: a Canadian Association of Internes and Residents well-being survey examining resident physician health and satisfaction within and outside of residency training in Canada. BMC Research Notes 2008;1(1):105. doi: https://doi.org/10.1186/1756-0500-1-105.

174. Manusov EG, Carr RJ, Rowane M, et al. Dimensions of happiness: a qualitative study of family practice residents. Journal of the American Board Family Practice 1995;8(5):367-75. Available from: https://www.jabfm.org/content/jabfp/8/5/367.full.pdf. (accessed 17/01/2022).

175. Goitein L, Shanafelt TD, Wipf JE, et al. The effects of work-hour limitations on resident well-being, patient care, and education in an internal medicine residency program. Arch Intern Med 2005;165(22):2601-6. doi: https://doi.org/10.1001/archinte.165.22.2601.

176. Fuss I, Nübling M, Hasselhorn HM, et al. Working conditions and Work-Family Conflict in German hospital physicians: psychosocial and organisational predictors and consequences. BMC Public Health 2008;8:353. doi: https://doi.org/10.1186/1471-2458-8-353.

177. Fischer J, Kumar S, Hatcher S. What makes psychiatry such a stressful profession? A qualitative study. Australas Psychiatry 2007;15(5):417-21. doi: https://doi.org/10.1080/10398560701439699.

178. Grol R, Mokkink H, Smits A, et al. Work satisfaction of general practitioners and the quality of patient care. Family practice 1985;2(3):128-35. doi: https://doi.org/10.1093/fampra/2.3.128.

179. San-Martín M, Delgado-Bolton R, Vivanco L. Professionalism and occupational well-being: Similarities and differences among Latin American health professionals. Frontiers in Psychology 2017;8. doi: https://doi.org/10.3389/fpsyg.2017.00063.

180. Bourne T, Shah H, Falconieri N, et al. Burnout, well-being and defensive medical practice among obstetricians and gynaecologists in the UK: cross-sectional survey study. BMJ Open 2019;9:e030968. doi: http://dx.doi.org/10.1136/bmjopen-2019-030968.

181. Alexandrova-Karamanova A, Todorova I, Montgomery A, et al. Burnout and health behaviors in health professionals from seven European countries. International archives of occupational and environmental health 2016;89(7):1059-75. doi: https://dx.doi.org/10.1007/s00420-016-1143-5.

182. Bragard I, Dupuis G, Razavi D, et al. Quality of work life in doctors working with cancer patients. Occupational medicine (Oxford, England) 2012;62(1):34-40. doi: https://dx.doi.org/10.1093/occmed/kqr149.

183. Carmel S. The Professional Self-esteem of Physicians Scale, structure, properties, and the relationship to work outcomes and life satisfaction. Psychological reports 1997;80(2):591-602. doi: https://doi.org/10.2466/pr0.1997.80.2.591.

184. Hills D, Joyce CM. Workplace aggression in clinical medical practice: associations with job satisfaction, life satisfaction and self-rated health. The Medical journal of Australia, 2014;201(9):535-40. doi: https://doi.org/10.5694/mja13.00152.

185. Moller AC, Jager AJ, Williams GC, et al. US Physicians' Work Motivation and Their Occupational Health: A National Survey of Practicing Physicians. Medical Care 2019;57(5):334-40. doi: http://dx.doi.org/10.1097/MLR.0000000000001101.

186. Ratanawongsa N, Korthuis PT, Saha S, et al. Clinician Stress and Patient-Clinician Communication in HIV Care. Journal of general internal medicine 2012:1-8. doi: http://dx.doi.org/10.1007/s11606-012-2157-7.

187. Sangi-Haghpeykar H, Ambani DS, Carson SA. Stress, workload, sexual well-being and quality of life among physician residents in training. International journal of clinical practice 2009;63(3):462-7. doi: https://dx.doi.org/10.1111/j.1742-1241.2008.01845.x.

188. Sauerteig SO, Wijesuriya J, Tuck M, et al. Doctors' health and wellbeing: at the heart of the NHS's mission or still a secondary consideration? International Review of Psychiatry 2019. doi: http://dx.doi.org/10.1080/09540261.2019.1586165.

189. Shearer S, Toedt M. Family physicians' observations of their practice, well being, and health care in the United States. The Journal of family practice 2001;50(9):751-6.

190. Thomsen S, Dallender J, Soares J, et al. Predictors of a healthy workplace for Swedish and English psychiatrists. The British Journal of Psychiatry 1998;173:80-84. doi: https://doi.org/10.1192/bjp.173.1.80.

191. Tong SC, Tin AS, Tan DMH, et al. The health-related quality of life of junior doctors. Annals of the Academy of Medicine Singapore 2012;41(10):444-50. Available from: https://annals.edu.sg/pdf/41VolNo10Oct2012/V41N10p444.pdf. (accessed 17/01/2022).

192. Veronese G, Pepe A. Sense of Coherence as a Determinant of Psychological Well-Being Across Professional Groups of Aid Workers Exposed to War Trauma. Journal of interpersonal violence 2017;32(13):1899-920. doi: http://dx.doi.org/10.1177/0886260515590125.

193. Waddimba AC, Scribani M, Hasbrouck MA, et al. Resilience among employed physicians and mid‐level practitioners in upstate New York. Health Services Research 2016;51(5):1706-34. doi: https://doi.org/10.1111/1475-6773.12499.

194. Zare SM, Galanko J, Behrns KE, et al. Psychological well-being of surgery residents before the 80-hour work week: A multiinstitutional study. Journal of the American College of Surgeons 2004;198(4):633-40. doi: http://dx.doi.org/10.1016/j.jamcollsurg.2003.10.006.

195. Lemaire JB, Wallace JE. How physicians identify with predetermined personalities and links to perceived performance and wellness outcomes: a cross-sectional study. BMC Health Services Research 2014;14(1):616. doi: https://doi.org/10.1186/s12913-014-0616-z.

196. Hoonpongsimanont W, Murphy M, Kim CH, et al. Emergency medicine resident well-being: stress and satisfaction. Occupational Medicine 2014;64(1):45-8. doi: https://doi.org/10.1093/occmed/kqt139.

197. Blazevska Stoilkovska B, Surbanovska O, Fritzhand A, et al. Life role salience and subjective well-being among Macedonian employees: Does family-supportive organization perception moderate this relationship. International journal of occupational medicine and environmental health 2018;31(3):281-91. doi: http://dx.doi.org/10.13075/ijomeh.1896.01204.

198. Ding M, Babenko O, Koppula S, et al. Physicians as Teachers and Lifelong Learners. The Journal of continuing education in the health professions 2019;39(1):2-6. doi: http://dx.doi.org/10.1097/CEH.0000000000000228.

199. Klein J, Frie KG, Blum K, et al. Psychosocial stress at work and perceived quality of care among clinicians in surgery. BMC Health Services Research 2011;11:109. doi: http://dx.doi.org/10.1186/1472-6963-11-109.

200. Mazotti LA, Vidyarthi AR, Wachter RM, et al. Impact of duty-hour restriction on resident inpatient teaching. Journal of hospital medicine 2009;4(8):476-80. doi: https://dx.doi.org/10.1002/jhm.448.

201. Olson K, Kemper KJ. Factors associated with well-being and confidence in providing compassionate care. Journal of evidence-based complementary & alternative medicine 2014;19(4):292-6. doi: https://dx.doi.org/10.1177/2156587214539977.

202. Salmoirago-Blotcher E, Fitchett G, Leung K, et al. An exploration of the role of religion/spirituality in the promotion of physicians' wellbeing in Emergency Medicine. Preventive Medicine Reports 2016;3:189-95. doi: http://dx.doi.org/10.1016/j.pmedr.2016.01.009.

203. Trockel MT, Hamidi MS, Menon NK, et al. Self-valuation: Attending to the Most Important Instrument in the Practice of Medicine. Mayo Clinic Proceedings, 2019;94(10):2022-31. doi: http://dx.doi.org/10.1016/j.mayocp.2019.04.040.

204. Turnell A, Rasmussen V, Butow P, et al. An exploration of the prevalence and predictors of work-related well-being among psychosocial oncology professionals: An application of the job demands-resources model. Palliative & supportive care 2016;14(1):33-41. doi: http://dx.doi.org/10.1017/S1478951515000693.

205. Grunfeld E, Zitzelsberger L, Coristine M, et al. Job stress and job satisfaction of cancer care workers. Psychooncology 2005;14(1):61-9.

206. Prins JT, Hoekstra-Weebers JE, Gazendam-Donofrio SM, et al. Burnout and engagement among resident doctors in the Netherlands: a national study. Medical Education 2010;44(3):236-47. doi: https://doi.org/10.1111/j.1365-2923.2009.03590.x.

207. Lambden JP, Chamberlin P, Kozlov E, et al. Association of Perceived Futile or Potentially Inappropriate Care With Burnout and Thoughts of Quitting Among Health-Care Providers. The American journal of hospice & palliative care 2018:1049909118792517. doi: http://dx.doi.org/10.1177/1049909118792517.

208. Salles A, Milam L, Cohen G, et al. The relationship between perceived gender judgment and well-being among surgical residents. American Journal of Surgery 2018;215(2):233-37.

209. Lall MD, Gaeta TJ, Chung AS, et al. Assessment of physician well-being, part one: Burnout and other negative states. Western Journal of Emergency Medicine 2019;20(2):278-90. doi: http://dx.doi.org/10.5811/westjem.2019.1.39665.

210. Lall MD, Gaeta TJ, Chung AS, et al. Assessment of physician well-being, part two: Beyond burnout. Western Journal of Emergency Medicine 2019;20(2):291-304. doi: http://dx.doi.org/10.5811/westjem.2019.1.39666.

211. Shanafelt TD, Kaups KL, Nelson H, et al. An interactive individualized intervention to promote behavioral change to increase personal well-being in US surgeons. Annals of Surgery 2014;259(1):82-8. doi: https://dx.doi.org/10.1097%2FSLA.0b013e3182a58fa4.

212. Wen LS, Baca JT, O'Malley P, et al. Implementation of small-group reflection rounds at an emergency medicine residency program. Canadian Journal of Emergency Medicine 2013;15(3):175-7. doi: https://doi.org/10.2310/8000.2013.130935.

213. Hurst C, Kahan D, Ruetalo M, et al. A year in transition: a qualitative study examining the trajectory of first year residents' well-being. BMC medical education 2013;13:96. doi: https://doi.org/10.1186/1472-6920-13-96.

214. Andolsek KM. Physician Well-Being: Organizational Strategies for Physician Burnout. FP essentials 2018;471:20-24.

215. Brady KJS, Kazis LE, Sheldrick RC, et al. Selecting physician well-being measures to assess health system performance and screen for distress: Conceptual and methodological considerations. Current Problems in Pediatric and Adolescent Health Care 2019:100662. doi: http://dx.doi.org/10.1016/j.cppeds.2019.100662.

216. Saadat H, Kain ZN. Wellness interventions for anesthesiologists. Current Opinion in Anaesthesiology 2018;31(3):375-81. doi: http://dx.doi.org/10.1097/ACO.0000000000000598.

217. Trockel M, Bohman B, Lesure E, et al. A brief instrument to assess both burnout and professional fulfillment in physicians: Reliability and validity, including correlation with self-reported medical errors, in a sample of resident and practicing physicians. Academic Psychiatry 2018;42(1):11-24. doi: https://doi.org/10.1007/s40596-017-0849-3.

218. Chung A, Battaglioli N, Lin M, et al. JGME-ALiEM Hot Topics in Medical Education: An Analysis of a Virtual Discussion on Resident Well-Being. Journal of graduate medical education 2018;10(1):36-42. doi: http://dx.doi.org/10.4300/JGME-D-17-00475.1.

219. Elovainio M, Heponiemi T, Kuusio H, et al. Developing a short measure of organizational justice: A multisample health professionals study. Journal of Occupational and Environmental Medicine 2010;52(11):1068-74. doi: http://dx.doi.org/10.1097/JOM.0b013e3181f8447c.

220. Fisher J. Development and application of a spiritual well-being questionnaire called SHALOM. Religions 2010;1(1):105-21. doi: https://doi.org/10.3390/rel1010105.

221. Forbes MP, Iyengar S, Kay M. Barriers to the psychological well-being of Australian junior doctors: A qualitative analysis. BMJ Open 2019;9(6):e027558. doi: http://dx.doi.org/10.1136/bmjopen-2018-027558.

222. Steven A, Oxley J, Fleming WG. Mentoring for NHS doctors: perceived benefits across the personal-professional interface. Journal of the Royal Society of Medicine 2008;101(11):552-7. doi: https://dx.doi.org/10.1258/jrsm.2008.080153.

223. Horwitz N, Bascunan L, Schiattino I, et al. Generation and validation of a scale to measure physicians' wellbeing. Revista Medica de Chile 2010;138(9):1084-90. doi: https://dx.doi.org/S0034-98872010000900002.

224. Fang CK, Li PY, Lai ML, et al. Establishing a 'Physician's Spiritual Well-being Scale' and testing its reliability and validity. Journal of Medical Ethics 2011;37(1):6-12. doi: https://doi.org/10.1136/jme.2010.037200 (accessed Jan).

225. Drks. Otium in the hospital? A mindfulness-based intervention for resident physicians. http://www.who.int/trialsearch/Trial2.aspx?TrialID=DRKS00014015 2018.

226. Bourne T, De Cock B, Wynants L, et al. Doctors' perception of support and the processes involved in complaints investigations and how these relate to welfare and defensive practice: A cross-sectional survey of the UK physicians. BMJ Open 2017;7(11):e017856. doi: http://dx.doi.org/10.1136/bmjopen-2017-017856.

227. Bruno A, Bracco F. Promoting safety through well-being: An experience in healthcare. Frontiers in Psychology, 2016;7. doi: https://doi.org/10.3389/fpsyg.2016.01208.

228. Degen C, Li J, Angerer P. Physicians' intention to leave direct patient care: an integrative review. Human resources for health 2015;13:74. doi: http://dx.doi.org/10.1186/s12960-015-0068-5.

229. Desai SV, Asch DA, Bellini LM, et al. Education Outcomes in a Duty-Hour Flexibility Trial in Internal Medicine. New England journal of medicine 2018;378(16):1494‐508.

230. Fletcher KE, Underwood W, III, Davis SQ, et al. Effects of Work Hour Reduction on Residents' Lives: A Systematic Review. JAMA: Journal of the American Medical Association 2005;294(9):1088-100. doi: https://doi.org/10.1001/jama.294.9.1088.

231. Howard R, Kirkley C, Baylis N. Personal resilience in psychiatrists: Systematic review. BJPsych Bulletin 2019;43(5):209-15. doi: http://dx.doi.org/10.1192/bjb.2019.12.

232. Klein M, Andersen LPH, Alamili M, et al. Psychological and physical stress in surgeons operating in a standard or modern operating room. Surgical laparoscopy, endoscopy & percutaneous techniques 2010;20(4):237-42.

233. Ziegler S, Merker H, Schmid M, et al. The impact of the inpatient practice of continuous deep sedation until death on healthcare professionals' emotional well-being: A systematic review. BMC Palliative Care 2017;16(1):30. doi: http://dx.doi.org/10.1186/s12904-017-0205-0.

234. Robieux L, Karsenti L, Pocard M, et al. Let's talk about empathy! Patient Education and Counseling 2018;101(1):59-66. doi: http://dx.doi.org/10.1016/j.pec.2017.06.024.

235. Raj KS. Well-Being in Residency: A Systematic Review. Journal of graduate medical education 2016;8(5):674-84. doi: http://dx.doi.org/10.4300/JGME-D-15-00764.1.

236. Scheepers RA, Boerebach BC, Arah OA, et al. A Systematic Review of the Impact of Physicians' Occupational Well-Being on the Quality of Patient Care. Int J Behav Med 2015;22(6):683-98. doi: https://dx.doi.org/10.1007%2Fs12529-015-9473-3.

237. Murray M, Murray L, Donnelly M. Systematic review of interventions to improve the psychological well-being of general practitioners. BMC family practice 2016;17:36. doi: http://dx.doi.org/10.1186/s12875-016-0431-1.

238. West CP, Dyrbye LN, Erwin PJ, et al. Interventions to prevent and reduce physician burnout: a systematic review and meta-analysis. Lancet (London, England) 2016;388(10057):2272-81. doi: https://dx.doi.org/10.1016/S0140-6736(16)31279-X.

239. Kelm Z, Womer J, Walter JK, et al. Interventions to cultivate physician empathy: a systematic review. BMC medical education 2014;14:219. doi: http://dx.doi.org/10.1186/1472-6920-14-219.

240. Maslach C, Jackson SE. The measurement of experienced burnout. Journal of organizational behavior 1981;2(2):99-113.

241. Young AE, Brookes ST, Avery KNL, et al. A systematic review of core outcome set development studies demonstrates difficulties in defining unique outcomes. Journal of Clinical Epidemiology 2019;115:14-24. doi: https://doi.org/10.1016/j.jclinepi.2019.06.016.

242. Degen C, Li J, Angerer P. Physicians' intention to leave direct patient care: an integrative review. Human resources for health 2015;13:74.

243. Fletcher KE, Underwood W, III, Davis SQ, et al. Effects of Work Hour Reduction on Residents' Lives: A Systematic Review. JAMA: Journal of the American Medical Association 2005;294(9):1088-100.

244. Howard R, Kirkley C, Baylis N. Personal resilience in psychiatrists: Systematic review. BJPsych Bulletin 2019;43(5):209-15.

245. Murray M, Murray L, Donnelly M. Systematic review of interventions to improve the psychological well-being of general practitioners. BMC family practice 2016;17:36.

246. Raj KS. Well-Being in Residency: A Systematic Review. Journal of graduate medical education 2016;8(5):674-84.

247. Scheepers RA, Boerebach BC, Arah OA, et al. A Systematic Review of the Impact of Physicians' Occupational Well-Being on the Quality of Patient Care. Int J Behav Med 2015;22(6):683-98.

248. West CP, Dyrbye LN, Erwin PJ, et al. Interventions to prevent and reduce physician burnout: a systematic review and meta-analysis. Lancet (London, England) 2016;388(10057):2272-81.

249. Kelm Z, Womer J, Walter JK, et al. Interventions to cultivate physician empathy: a systematic review. BMC medical education 2014;14:219.

250. Adobe Acrobat Pro DC Version 2021.007.20099. [program]: Adobe Inc, 2021.

251. Cooke P J MPT, Connor k. Measuring Well-Being: A Review of Instruments. The Counseling Psychologist 2016;44(5):730-57.

252. Page MJ, McKenzie JE, Bossuyt PM, et al. The PRISMA 2020 statement: an updated guideline for reporting systematic reviews. Syst Rev 2021;10(1):89.

253. Kelm DJ, Ridgeway JL, Gas BL, et al. Mindfulness meditation and interprofessional cardiopulmonary resuscitation: A mixed-methods pilot study. Teaching and Learning in Medicine 2018. doi: https://doi.org/10.1080/10401334.2018.1462186.

254. Tawfik DS, Profit J, Morgenthaler TI, et al. Physician Burnout, Well-being, and Work Unit Safety Grades in Relationship to Reported Medical Errors. Mayo Clin Proc 2018;93(11):1571-80. doi: http://dx.doi.org/10.1016/j.mayocp.2018.05.014.

255. Lian YL, Liu JW, Zhang C, et al. Comparison of strain of mental workers by characteristics. Chinese journal of industrial hygiene and occupational diseases 2009;27(12):725-29.
